# Mental health help-seeking among young people with out-of-home care experience: a mixed-methods systematic review and meta-analysis protocol

**DOI:** 10.1136/bmjopen-2026-118618

**Published:** 2026-07-20

**Authors:** Willem Stander, Emma Rebecca Wallace, Jolie Keemink, Omar Mohamed, Jason Schaub

**Affiliations:** 1 School of Psychology, University of Birmingham, Birmingham, UK; 2 Centre for Health Services Studies, School of Social Sciences, University of Kent, Canterbury, UK; 3 Brent Council, Wembley, UK; 4 School for Policy Studies, University of Bristol, Bristol, UK

**Keywords:** Adolescent, Young Adult, Health Equity, MENTAL HEALTH, Health Services Accessibility, Vulnerable Populations

## Abstract

**Abstract:**

**Introduction:**

Out-of-home care experienced young people have elevated mental health needs, yet their mental health help-seeking remains insufficiently understood. Understanding help-seeking is critical for identifying barriers and facilitators that shape whether, how and from whom care-experienced young people seek and access mental health support. Existing syntheses are limited in scope and have under-represented quantitative evidence, residential care, care leavers and equity-relevant subgroups. This review will synthesise international evidence on sources, barriers and facilitators of mental health help-seeking among care-experienced young people.

**Methods and analysis:**

This mixed-methods systematic review and meta-analysis protocol is reported in accordance with Preferred Reporting Items for Systematic Reviews and Meta-Analyses Protocols and informed by Preferred Reporting Items for Systematic Review and Meta-Analyses-Equity. Electronic searches will be undertaken in PsycINFO, MEDLINE, EMBASE, Applied Social Sciences Index and Abstracts, Social Sciences Citation Index, Social Policy and Practice, Health Management Information Consortium, ProQuest Dissertations & Theses Global, Cumulative Index to Nursing and Allied Health Literature (CINAHL) Plus and Child Development and Adolescent Studies, alongside grey literature and citation searching. Eligible studies will include qualitative, quantitative and mixed-methods research involving care-experienced young people aged 13–25 years, including care leavers, and studies reporting help-seeking initiated by young people or on their behalf by adults, carers or professionals. Quantitative and qualitative evidence will be synthesised separately before narrative integration. Meta-analysis will be considered where independent quantitative studies report sufficiently comparable populations, outcomes and effect measures; otherwise, findings will be synthesised narratively using Synthesis Without Meta-analysis (SWiM) guidance. Qualitative findings will be synthesised using thematic synthesis. Study quality will be appraised using the Newcastle–Ottawa Scale, Critical Appraisal Skills Programme and Mixed Methods Appraisal Tool. Equity-relevant characteristics will be extracted where reported, informed by the PROGRESS-Plus framework. Care-experienced young advisors have informed the wider programme and this review, and will contribute to interpretation and dissemination.

**Ethics and dissemination:**

Ethical approval is not required as the review involves secondary analysis of the existing evidence base. Findings will be disseminated through peer-reviewed publication, conferences, webinars and accessible outputs co-developed with care-experienced young people.

**PROSPERO registration number:**

CRD420251047424.

**STRENGTHS AND LIMITATIONS OF THIS STUDY**

- This mixed-methods review will use segregated synthesis to integrate qualitative, quantitative and mixed-methods evidence.
- Searches will cover health, psychology, social science, social policy, education, nursing and grey literature databases.
- Screening, data extraction and quality appraisal will be undertaken independently by two reviewers.
- Preferred Reporting Items for Systematic Review and Meta-Analyses-Equity and PROGRESS-Plus framework will guide extraction and synthesis of equity-relevant data.
- English-language limits, heterogeneous methods and international variation in care systems may affect transferability.

## Introduction

 Adolescence and early adulthood are periods of heightened vulnerability, with the highest incidence of common mental health problems observed across the life course.^1^,^3^ International evidence indicates that levels of psychological distress and diagnosable mental health conditions among young people have increased in recent decades.^4^,^6^ Within this broader context, young people with experience of out-of-home care (OOHC; hereafter referred to as care-experienced young people) face substantially elevated and often complex mental health needs. OOHC arrangements include non-biological foster care, kinship care, residential or group care, and supported or independent living.^7 8^ Although OOHC systems, legal frameworks and service configurations vary internationally, these care settings share common features across jurisdictions. Many young people enter care following exposure to adversity and trauma, their lives are shaped by statutory child welfare systems and formal support frequently ends at age-based thresholds rather than according to need.^8 9^ OOHC is therefore a policy and practice priority internationally, with cross-national efforts underway to improve outcomes for care-experienced young people.^10^

A substantial body of evidence demonstrates that care-experienced young people experience disproportionately high rates of mental health difficulties compared with their peers. Across studies, estimates suggest that 30%–80% of care-experienced young people meet the criteria for at least one diagnosable mental health condition, with higher levels of comorbidity and suicidality than in the general population.^11^,^17^ These inequalities are shaped by early adversities, including abuse, neglect, family disruption and socioeconomic disadvantage, and may be further compounded by experiences within the care system, such as placement instability, separation from siblings and frequent changes in caregivers.^18 19^ Over the life course, care-experienced individuals are also more likely to experience poorer physical health, lower educational attainment, housing insecurity, reduced employment opportunities and contact with the criminal justice system,^20^,^23^ contributing to cumulative and enduring mental health inequalities.^24^

Despite elevated levels of need, mental health help-seeking among care-experienced young people remains insufficiently understood. Help-seeking is conceptualised as an active coping process involving efforts to obtain external assistance—such as information, advice, treatment or emotional support—from formal or professional sources (eg, mental health professionals, primary care providers, social workers, school-based services), and informal sources (eg, family members, peers, partners).^25 26^ It is a dynamic, multi-stage process that encompasses attitudes and intentions as well as behaviours, beginning with symptom recognition and appraisal of need, followed by decisions about whether and where to seek help, and attempts to access and engage with sources of support.^25 26^ Service utilisation represents one possible outcome within this pathway, referring specifically to contact with formal services, while help-seeking more broadly captures earlier decision-making stages and a wider range of supports, shaped by perceptions of need, stigma, service availability, and wider social and structural conditions. This broader focus is critical in OOHC contexts, where help-seeking opportunities and pathways are often mediated by adults and statutory systems, allowing distinction between young people’s own help-seeking agency and service contact shaped by statutory or organisational decision-making.^27^

Drawing on both previous evidence syntheses and primary empirical studies, emerging evidence suggests that help-seeking and uptake of mental health support among care-experienced young people is often disproportionate to their level of need.^27 28^ While mental health support may be offered when young people enter care, engagement is frequently not sustained, and a substantial proportion do not access support at all.^29^ Reported barriers include stigma, concerns about privacy and confidentiality, limited trust in professionals, perceived lack of understanding of lived experience, difficulties navigating services and restricted involvement in decision-making.^27 30 31^ In addition, research has identified systemic barriers that may restrict help-seeking and access to mental health support, including placement type, placement instability and the frequency of placement changes.^29 32^ Consistent with patterns in the general youth population, when support is sought, care-experienced young people often report a preference for informal sources, such as help from family members (biological or within OOHC), friends or romantic partners.^27 29 33 34^ Understanding these barriers and facilitators is critical for designing policies, services and interventions that support care-experienced young people to seek and access mental health support.^30^

A previous international systematic review provides valuable but incomplete insight into mental health help-seeking among care-experienced young people, particularly with respect to variation across OOHC contexts and marginalised subgroups within this population. Powell *et al*^27^ focused predominantly on qualitative evidence and selected OOHC settings (foster or kinship care), excluding quantitative studies, grey literature, young people in residential care and care leavers. These limitations are important because young people in residential care and those transitioning out of care consistently report some of the highest levels of mental health need and may face distinct and compounded barriers to help-seeking.^2335^,^40^ Young people in residential care often have more complex needs and greater placement instability, while care leavers must navigate mental health support following care exit, when transitions to adult services coincide with reduced statutory support.^14 35 39 41^

In addition, the existing evidence base has rarely examined how mental health help-seeking varies across equity-relevant social and structural characteristics within care-experienced populations.^14 27^ Care-experienced young people are disproportionately represented among groups already exposed to structural disadvantage and discrimination, including minoritised ethnic groups and lesbian, gay, bisexual, transgender and queer or questioning (LGBTQ+) communities,^42^,^44^ and many experience disability or neurodevelopmental differences.^45 46^ Evidence from the general population indicates that these groups often face additional barriers to accessing mental health support,^47^,^49^ suggesting the potential for compounded disadvantage among some care-experienced young people.^19 29 42 50^ Reviews, primary studies and policy documents also highlight inconsistent reporting of equity-relevant characteristics in OOHC research, limiting understanding of how these factors shape mental health needs and help-seeking.^51^,^54^ This reflects a broader limitation in systematic reviews, which often insufficiently address equity considerations, reducing their capacity to inform policy and practice.^55^ An equity-oriented approach is therefore needed to avoid obscuring variation within care-experienced populations and to improve relevance for those most at risk of unmet need.

At present, no comprehensive international synthesis integrates qualitative, quantitative and mixed-methods evidence on mental health help-seeking among care-experienced young people across OOHC contexts and equity-relevant characteristics. This review will synthesise evidence on sources of support sought; contextual influences on help-seeking (including individual, care-related, organisational and structural influences); and barriers and facilitators shaping access to support. Guided by an equity-informed approach using the PROGRESS-Plus framework,^56^ this review will examine variation across care contexts and social positions to inform policy, practice and intervention development aimed at improving timely and equitable access to mental health support for care-experienced young people.

This international systematic review will address the following questions:

1. What formal and informal sources of mental health support do care-experienced young people seek when experiencing mental health problems?
2. What contextual factors (including individual, relational, care-related, organisational and structural factors) are associated with variation in mental health help-seeking among care-experienced young people?
3. What barriers and facilitators to mental health help-seeking are reported, and how do these shape access to mental health support across care contexts and equity-relevant groups?

## Methods and analysis

### Protocol registration and reporting standards

This protocol has been registered with the PROSPERO international prospective register for systematic reviews (registration number: CRD420251047424) and is reported in accordance with the Preferred Reporting Items for Systematic Reviews and Meta-Analyses Protocols (PRISMA-P) guidelines^57 58^ (see online supplemental appendix 1). Prior to registration, we searched PROSPERO, the Cochrane Database of Systematic Reviews and the Open Science Framework (OSF) to identify ongoing or completed reviews on this topic and found no reviews with an overlapping scope. A systematic review approach was selected, rather than a scoping review, to enable critical appraisal and synthesis of evidence and assessment of confidence in findings, rather than mapping the literature alone.^59^ The final review will be reported following the Preferred Reporting Items for Systematic Review and Meta-Analyses (PRISMA) statement and the equity extension of the PRISMA statement, PRISMA-E 2012.^60^,^62^ Any amendments to the protocol, including those made during peer review, will be documented in PROSPERO and published with the results of the review.

### Eligibility criteria (PICOS)

Eligibility criteria were defined using the Population, Intervention/Exposure, Comparator, Outcome and Study Design (PICOS) framework.

#### Population

Studies involving care-experienced young people aged 13–25 years will be included. Care-experienced is defined as individuals currently living in, or who have previously lived in, statutory OOHC. OOHC refers to placement outside the birth family on a voluntary or compulsory basis, including non-biological foster care, formal kinship care, residential or group care, and supported accommodation or semi-independent/independent living arrangements provided as part of statutory care-leaving pathways. This definition aligns closely with the UK concept of looked-after children, derived from the Children Act 1989 and the Children (Scotland) Act 1995, and shares common features with statutory OOHC systems internationally.^63 64^ Time in care is not restricted. Excluded populations include general population samples; in-home care arrangements (ie, voluntary family support without removal); care without specified statutory involvement (eg, informal kinship care); and adoption (as post-adoption support pathways and legal status differ substantially from statutory OOHC).

We selected this age range as early adolescence marks the stage at which mental health difficulties commonly emerge and help-seeking intentions and behaviours can be observed and reported, while autonomy and participation in health and care decision-making increase^3465^,^67^; inclusion to age 25 aligns with statutory support for care leavers in several jurisdictions, including the UK. Studies including wider age ranges will be eligible where data for participants aged 13–25 years can be disaggregated, or where most participants are within this age range, defined as a majority (>50%) where this can be determined. Where age data are incomplete, studies may still be included if they are clearly adolescent-focused or young-person-focused, for example, through the sample description, recruitment setting, eligibility criteria, or reported median/mean age, and findings are directly relevant to mental health help-seeking. These studies will be flagged during extraction and excluded from age-specific quantitative synthesis. Where age relevance remains unclear, authors will be contacted where feasible. Studies involving carers, professionals or other adults will be eligible where findings explicitly relate to the mental health help-seeking processes of care-experienced young people who meet, or can be reasonably assessed against, these age criteria.

#### Exposure/phenomenon of interest

The exposure of interest is mental health help-seeking among care-experienced young people. This includes attitudes, intentions, decision-making and behaviours related to seeking support from formal/professional services and/or informal sources, as well as barriers, facilitators and interventions designed to support or improve help-seeking. Studies may address help-seeking related to diagnosed mental health conditions (eg, depression, anxiety, post-traumatic stress, bipolar disorder) and non-diagnostic mental health presentations (eg, psychological distress), including self-harm and suicidality, which are commonly studied as drivers or triggers of help-seeking irrespective of formal diagnosis. Studies focused solely on help-seeking for substance use, substance use disorders, substance use treatment, neurodiversity, learning disability, personality-related difficulties, educational support, disability services, or behavioural management will be excluded unless mental health help-seeking is explicitly examined. Where these issues are reported as co-occurring issues, barriers, facilitators or contextual influences shaping mental health help-seeking, they will be extracted and considered in the synthesis. Service utilisation will be included only where examined as part of the help-seeking process or pathway. Studies focused solely on treatment effectiveness, clinical outcomes or service utilisation (eg, service contact or uptake) without examining help-seeking will be excluded.

#### Comparison

Any comparator or none.

#### Outcomes

Help-seeking outcomes and review phenomena will include:

- help-seeking attitudes, intentions and behaviours, including recognition and appraisal of need, decisions about whether and where to seek help, and whether support was sought by young people themselves or initiated, facilitated or mediated by adults, carers or professionals;
- sources and patterns of formal and/or informal help sought;
- experiences and views of seeking and attempting to access mental health support;
- barriers and facilitators to seeking mental health support (barriers defined as factors that delay or prevent help-seeking; facilitators as factors that enable or encourage it); and,
- variation in help-seeking across OOHC contexts and equity-relevant characteristics.

Where possible, attitudes, intentions and behaviours will be extracted and reported separately, while recognition, appraisal and decision-making will be examined as components of the help-seeking process.

#### Study design and setting

Empirical qualitative, quantitative and mixed-methods studies reporting primary findings will be included, comprising peer-reviewed publications and relevant grey literature (eg, dissertations, theses, organisational and service reports). Inclusion of grey literature aims to capture practice-based evidence and lived-experience insights often under-represented in academic publications and to reduce publication bias.^68 69^ Commentaries, editorials and non-empirical reports will be excluded.

Studies will not be excluded based on methodological quality or reporting of equity-relevant characteristics; these factors will instead inform interpretation of findings. All included studies will be critically appraised (see Quality Appraisal).

Studies from any geographic setting will be eligible. Variation in national policy contexts, legal definitions of care and service structures will be documented and considered analytically as contextual influences on help-seeking and interpretation of findings.

No restrictions will be placed on year of publication. Only English-language publications will be included due to resource constraints.

### Information sources and search strategy

The following databases will be searched: PsycINFO, MEDLINE, EMBASE, Applied Social Sciences Index and Abstracts (ASSIA), Social Sciences Citation Index (SSCI), Social Policy and Practice, Health Management Information Consortium (HMIC), ProQuest Dissertations & Theses Global, Cumulative Index to Nursing and Allied Health Literature (CINAHL) Plus, and Child Development and Adolescent Studies.

The search strategy was developed in collaboration with an information scientist and piloted and refined to balance sensitivity and specificity. Searches will use a combination of controlled vocabulary terms (eg, Medical Subject Headings (MeSH)) and free-text keywords relating to (1) mental health, (2) help-seeking, (3) care-experienced populations and (4) young people. Search terms will be adapted for each database and searched in title, abstract, keyword/heading and subject fields as appropriate to each platform. Searches will be conducted from database inception to the final search date, with English-language limits applied where available. Reference lists of included studies and relevant reviews will be screened, and backward citation tracking undertaken, to identify additional eligible publications. The full search strategies for all databases, including limits applied, are provided in online supplemental appendix 2.

### Study selection

All identified records will be imported into Covidence and deduplicated. Titles and abstracts will be screened independently by two reviewers. Full-text articles of potentially eligible studies will be assessed independently by the same reviewers. Any disagreements at either stage will be resolved through discussion and, where necessary, consultation with another member of the research team.

### Data extraction

Data will be extracted using a review-specific form developed in Covidence and adapted from a previously published systematic review of young people’s mental health help-seeking.^34^ The form has been refined to capture OOHC context, conceptualisations and processes of help-seeking, experiences of support, and equity-relevant characteristics. Extracted information will include study characteristics (author, year, country), methodology and setting, participant characteristics (age range and key sociodemographic information) and OOHC context (eg, placement type, care leaver status). We will extract the mental health focus of each study and how help-seeking is defined or operationalised, including whether studies examine help-seeking attitudes, intentions or behaviours. Data will also include sources of support (formal and informal), recognition and appraisal of need, decisions about whether and where to seek help, whether help-seeking was self-initiated or mediated by adults or statutory systems, and reported experiences of seeking and attempting to access support. Key findings relating to barriers and facilitators to help-seeking will also be extracted. Where reported, we will also extract information on patient and public involvement (PPI) in study design, conduct or dissemination to descriptively assess the extent to which lived experience has informed help-seeking research in this field.

Equity-relevant characteristics will be extracted separately using the PROGRESS-Plus framework,^56^ a widely used approach for examining health inequalities. PROGRESS-Plus captures place of residence, race/ethnicity, occupation, gender/sex, religion, education, socioeconomic status and social capital, alongside additional characteristics associated with disadvantage (eg, disability, sexual orientation, gender identity and care status). These data will be used to examine variation in help-seeking where reported. While PROGRESS-Plus provides a structured approach to identifying equity characteristics, interpretation will remain dependent on the availability and quality of reporting in primary studies.^70^ Absence or incomplete reporting of equity characteristics will be treated as a substantive finding of the review.

The extraction form will be piloted independently by two reviewers on 10 included studies to assess clarity, completeness and consistency across qualitative, quantitative and mixed-methods evidence. Where possible, pilot studies will reflect methodological diversity. Refinements will be made prior to full data extraction. Following piloting, data from all included studies will be extracted independently by two reviewers. Discrepancies will be resolved through discussion and, where necessary, consultation with a third member of the review team. The level of extraction detail is considered feasible because pilot screening indicates a modest number of included studies, and the extraction form will be refined during piloting to ensure that only data relevant to the review questions are extracted.

### Quality appraisal

Appraisal will be conducted at the study level. Study quality will be assessed independently by two reviewers using design-appropriate critical appraisal tools, with disagreements resolved through discussion or consultation with a third reviewer. Given that previous systematic reviews of youth mental health help-seeking have predominantly identified observational quantitative and qualitative evidence,^31 34 71^ quantitative studies will be appraised using an adapted version of the Newcastle–Ottawa Scale (NOS),^72^ adapted for cross-sectional and other observational designs where appropriate. Mixed-methods studies will be appraised using the Mixed Methods Appraisal Tool (MMAT).^73^ Qualitative studies will be appraised using the Critical Appraisal Skills Programme (CASP) qualitative checklist.^74^ Because qualitative, quantitative and mixed-methods evidence will be synthesised separately before narrative integration, we selected design-appropriate tools for each evidence type. Quality appraisal will be used to inform interpretation of findings rather than as a criterion for study exclusion.

### Data analysis and synthesis

A segregated synthesis approach will be used, with qualitative and quantitative evidence synthesised separately and then integrated narratively at the interpretation stage. Quantitative findings will first be grouped, where possible, by help-seeking construct/process (including attitudes, intentions and behaviours), source of support (eg, formal or informal), population characteristics and mental health focus. Narrative synthesis is expected to be the primary approach because heterogeneity is anticipated across study designs, populations, measures and outcomes.^30 31 34^ Meta-analysis will be considered only where two or more independent quantitative studies report sufficiently comparable populations, outcomes and effect measures. Where feasible, this may include pooled estimates of help-seeking prevalence or proportions, associations between participant or care-context characteristics and help-seeking, or intervention effects. For dichotomous outcomes, we will extract or calculate ORs, risk ratios or proportions with 95% CIs; for continuous outcomes, we will extract means and SD and calculate mean differences or standardised mean differences where scales differ. Where pooling is appropriate, random-effects models will be used. Heterogeneity will be assessed using I², visual inspection of forest plots, and consideration of study-level differences in populations, outcomes and measures. Where pooling is inappropriate, findings will be synthesised narratively and reported using Synthesis Without Meta-analysis (SWiM) guidance.^75^ No formal subgroup analyses, sensitivity analyses or meta-regression are planned because of the anticipated heterogeneity and likely small number of comparable quantitative studies. Where data permit, variation in help-seeking will instead be explored narratively across population characteristics, care contexts and mental health focus.

Qualitative findings will be synthesised using inductive thematic synthesis, following Thomas and Harden.^76^ For qualitative and mixed-methods studies, relevant findings will be extracted in Covidence and coded separately from the extraction form using a qualitative analysis matrix. We will code relevant findings/results sections, including participant quotations, author-reported themes and subthemes, interpretive commentary, and any tables or conceptual models reporting qualitative findings relevant to mental health help-seeking. Background, methods and discussion sections will not be coded as primary synthesis data, but may be used to inform contextual interpretation and quality appraisal. Synthesis will involve line-by-line coding, development of descriptive themes, and generation of higher-order analytical themes related to help-seeking, barriers and facilitators. Coding will be conducted independently by two reviewers, with discrepancies resolved through discussion and refinement of the coding framework.

Findings from the quantitative and qualitative syntheses will then be integrated narratively to provide an integrated understanding of mental health help-seeking among care-experienced young people. Where data permit, variation in help-seeking patterns, barriers and facilitators will be explored by social and structural characteristics and across national policy and service contexts.

### Assessment of certainty/strength of evidence

Confidence in quantitative findings will be assessed independently by two reviewers using the Grading of Recommendations, Assessment, Development and Evaluation (GRADE) considerations,^77^ and confidence in qualitative findings using the Confidence in the Evidence from Reviews of Qualitative Research (GRADE-CERQual).^78^ Disagreements will be resolved through discussion or consultation with a third reviewer. Assessments will be conducted separately for each evidence stream and considered together during narrative integration. Data from mixed-methods studies will be incorporated into the relevant synthesis, with confidence assessed at the level of the synthesised findings.

Where quantitative synthesis is conducted, potential reporting biases will be assessed, including publication bias and selective reporting within studies, informed by quality appraisal and comparison of reported outcomes with study aims.

## Patient and public involvement

This protocol forms part of a wider programme of research on care-experienced young people’s mental health help-seeking and has been informed by PPI from the outset. Care-experienced young people contributed to the development of the wider project at the funding application stage, including shaping the research focus, equity considerations and proposed methods.

A young people’s advisory group comprising six care-experienced young people supports the wider research programme and has contributed through three structured meetings to date, including a research training session. Input from this group has informed the development of this review protocol, including the prioritisation of research questions and the emphasis on issues important to care-experienced young people, such as inequalities in access to support. Young advisors will continue to contribute to the interpretation of findings and dissemination activities, including helping to prioritise key considerations for reporting and practice recommendations.

A professional advisory group of academics and health and social care professionals with expertise in care-experienced populations and youth mental health will support the review at key stages, particularly interpretation and dissemination.

Consistent with best practice,^79^,^81^ advisory contributors will not be involved in technical quality appraisal using formal assessment tools; these assessments will be undertaken by the review team. PPI in the final review will be reported using the appropriate Guidance for Reporting Involvement of Patients and the Public 2 (GRIPP2) reporting checklist.^80^

## Limitations

Several methodological limitations are anticipated. Only English-language studies will be included, which may limit the review’s relevance to non-English-speaking regions and may under-represent evidence from non-Western contexts. Comparability and transferability of findings may also be limited by variation in how OOHC, care-leaving entitlements, mental health services and help-seeking are defined and organised across countries. Substantial heterogeneity is expected in study designs, populations, help-seeking measures and outcomes, which may limit the feasibility of meta-analysis. Although the review will use PRISMA-Equity and PROGRESS-Plus to guide extraction and synthesis of equity-relevant characteristics, analysis of inequalities will depend on the extent and quality of reporting in included studies.

## Ethics and dissemination

Ethical approval is not required as this study involves secondary analysis of the existing evidence base. Findings will be disseminated through one peer-reviewed systematic review publication, and conference and webinar presentations to academic, policy and practice audiences. Findings will also be used to inform free, accessible digital outputs co-developed with care-experienced young people for young people, professionals and policy audiences. Findings from this review will also inform the interpretation, contextualisation and dissemination of associated primary research within the wider programme of work on care-experienced young people’s mental health help-seeking, including an online survey and qualitative interviews with care-experienced young people.

## Supplementary material

10.1136/bmjopen-2026-118618 online supplemental file 1

10.1136/bmjopen-2026-118618 online supplemental file 2
