# Supplementary material for: Mental health help-seeking among young people with out-of-home care experience: a mixed-methods systematic review and meta-analysis protocol
Source: BMJ Open. 2026 Jul 20;16(7):e118618. doi: 10.1136/bmjopen-2026-118618 (PMC13386074; doi:10.1136/bmjopen-2026-118618)
Supplement: online supplemental file 2 [file bmjopen-16-7-s002.docx]

# **Supplementary Appendix 2: Full search strategies**

## **Ovid MEDLINE(R)**

**Platform:** Ovid
**Coverage:** From database inception to final date
**Limits:** English language

| **Line** | **Search terms** |
| --- | --- |
| 1 | Mental Health/ |
| 2 | Depression/ |
| 3 | Anxiety/ |
| 4 | Self-Injurious Behavior/ |
| 5 | (“mental health*” or “mental ill*” or “mental disorder*”).ti,ab. |
| 6 | (“psychological health*” or “psychological ill*” or “psychological disorder*”).ti,ab. |
| 7 | (depress* or anxi* or suicid* or “self-harm” or “self harm” or selfharm or “self injur*”).ti,ab. |
| 8 | 1 or 2 or 3 or 4 or 5 or 6 or 7 |
| 9 | help seeking behavior/ |
| 10 | (help-seeking or help seeking or helpseeking).ti,ab. |
| 11 | ((seek* or sought or seeking) adj3 (help or support or care or treatment)).ti,ab. |
| 12 | ((receiv* or get* or obtain*) adj3 (mental health adj3 (help or support or care or treatment))).ti,ab. |
| 13 | ((help-seeking or help seeking) adj3 (attitude* or intention* or behavio?r*)).ti,ab. |
| 14 | ((“barrier*” or “facilitator*”) adj3 (help-seeking or help seeking)).ti,ab. |
| 15 | ((access* or engag* or contact*) adj3 (mental health adj3 (service* or care or support))).ti,ab. |
| 16 | ((attitude* or perception*) adj5 (mental health adj5 (service* or care))).ti,ab. |
| 17 | 9 or 10 or 11 or 12 or 13 or 14 or 15 or 16 |
| 18 | exp Foster Home Care/ |
| 19 | exp Child, Foster/ |
| 20 | exp Residential Facilities/ |
| 21 | (“foster care” or “foster home care” or “foster child*” or “foster youth*”).ti,ab. |
| 22 | (“kinship care” or “relative care” or “kinship placement*”).ti,ab. |
| 23 | (“residential care” or “residential home*” or “children’s home*” or “group home*”).ti,ab. |
| 24 | (“out-of-home care” or “out of home care” or “looked after” or “looked-after”).ti,ab. |
| 25 | ((care adj2 leav*) or (care adj2 experienc*)).ti,ab. |
| 26 | (transition* adj3 care).ti,ab. |
| 27 | 18 or 19 or 20 or 21 or 22 or 23 or 24 or 25 or 26 |
| 28 | Adolescent/ |
| 29 | Young Adult/ |
| 30 | Child/ |
| 31 | (“young people” or “young person*” or “young adult*” or “emerging adult*” or adolescen* or youth or youths or teen* or child*).ti,ab. |
| 32 | 28 or 29 or 30 or 31 |
| 33 | 8 and 17 and 27 and 32 |
| 34 | limit 33 to english language |

## **APA PsycINFO**

**Platform:** Ovid
**Coverage:** From database inception to final date
**Limits:** English language

| **Line** | **Search terms** |
| --- | --- |
| 1 | Mental Health/ |
| 2 | Anxiety/ |
| 3 | Self-Injurious Behavior/ |
| 4 | (“mental health*” or “mental ill*” or “mental disorder*”).ti,ab. |
| 5 | (“psychological health*” or “psychological ill*” or “psychological disorder*”).ti,ab. |
| 6 | (depress* or anxi* or suicid* or “self-harm” or “self harm” or selfharm or “self injur*”).ti,ab. |
| 7 | 1 or 2 or 3 or 4 or 5 or 6 |
| 8 | help seeking behavior/ |
| 9 | (help-seeking or help seeking or helpseeking).ti,ab. |
| 10 | ((seek* or sought or seeking) adj3 (help or support or care or treatment)).ti,ab. |
| 11 | ((receiv* or get* or obtain*) adj3 (mental health adj3 (help or support or care or treatment))).ti,ab. |
| 12 | ((help-seeking or help seeking) adj3 (attitude* or intention* or behavio?r*)).ti,ab. |
| 13 | ((“barrier*” or “facilitator*”) adj3 (help-seeking or help seeking)).ti,ab. |
| 14 | ((access* or engag* or contact*) adj3 (mental health adj3 (service* or care or support))).ti,ab. |
| 15 | ((attitude* or perception*) adj5 (mental health adj5 (service* or care))).ti,ab. |
| 16 | 8 or 9 or 10 or 11 or 12 or 13 or 14 or 15 |
| 17 | Foster Care/ |
| 18 | Foster Children/ |
| 19 | Residential Care Institutions/ |
| 20 | Group Homes/ |
| 21 | Child Welfare/ |
| 22 | (“foster care” or “foster home care” or “foster child*” or “foster youth*”).ti,ab. |
| 23 | (“kinship care” or “relative care” or “kinship placement*”).ti,ab. |
| 24 | (“residential care” or “residential home*” or “children’s home*” or “group home*”).ti,ab. |
| 25 | (“out-of-home care” or “out of home care” or “looked after” or “looked-after”).ti,ab. |
| 26 | ((care adj2 leav*) or (care adj2 experienc*)).ti,ab. |
| 27 | (transition* adj3 care).ti,ab. |
| 28 | 17 or 18 or 19 or 20 or 21 or 22 or 23 or 24 or 25 or 26 or 27 |
| 29 | (“young people” or “young person*” or “young adult*” or “emerging adult*” or adolescen* or youth or youths or teen* or child*).ti,ab. |
| 30 | 7 and 16 and 28 and 29 |
| 31 | limit 30 to english language |

## **Embase**

**Platform:** Ovid
**Coverage:** From database inception to final date
**Limits:** English language

| **Line** | **Search terms** |
| --- | --- |
| 1 | Mental Health/ |
| 2 | Depression/ |
| 3 | Anxiety/ |
| 4 | Self-Injurious Behavior/ |
| 5 | (“mental health*” or “mental ill*” or “mental disorder*”).ti,ab. |
| 6 | (“psychological health*” or “psychological ill*” or “psychological disorder*”).ti,ab. |
| 7 | (depress* or anxi* or suicid* or “self-harm” or “self harm” or selfharm or “self injur*”).ti,ab. |
| 8 | 1 or 2 or 3 or 4 or 5 or 6 or 7 |
| 9 | help seeking behavior/ |
| 10 | (help-seeking or help seeking or helpseeking).ti,ab. |
| 11 | ((seek* or sought or seeking) adj3 (help or support or care or treatment)).ti,ab. |
| 12 | ((receiv* or get* or obtain*) adj3 (mental health adj3 (help or support or care or treatment))).ti,ab. |
| 13 | ((help-seeking or help seeking) adj3 (attitude* or intention* or behavio?r*)).ti,ab. |
| 14 | ((“barrier*” or “facilitator*”) adj3 (help-seeking or help seeking)).ti,ab. |
| 15 | ((access* or engag* or contact*) adj3 (mental health adj3 (service* or care or support))).ti,ab. |
| 16 | ((attitude* or perception*) adj5 (mental health adj5 (service* or care))).ti,ab. |
| 17 | 9 or 10 or 11 or 12 or 13 or 14 or 15 or 16 |
| 18 | exp Foster Home Care/ |
| 19 | exp Child, Foster/ |
| 20 | exp Residential Facilities/ |
| 21 | (“foster care” or “foster home care” or “foster child*” or “foster youth*”).ti,ab. |
| 22 | (“kinship care” or “relative care” or “kinship placement*”).ti,ab. |
| 23 | (“residential care” or “residential home*” or “children’s home*” or “group home*”).ti,ab. |
| 24 | (“out-of-home care” or “out of home care” or “looked after” or “looked-after”).ti,ab. |
| 25 | ((care adj2 leav*) or (care adj2 experienc*)).ti,ab. |
| 26 | (transition* adj3 care).ti,ab. |
| 27 | 18 or 19 or 20 or 21 or 22 or 23 or 24 or 25 or 26 |
| 28 | Adolescent/ |
| 29 | Young Adult/ |
| 30 | Child/ |
| 31 | (“young people” or “young person*” or “young adult*” or “emerging adult*” or adolescen* or youth or youths or teen* or child*).ti,ab. |
| 32 | 28 or 29 or 30 or 31 |
| 33 | 8 and 17 and 27 and 32 |
| 34 | limit 33 to english language |

## **HMIC Health Management Information Consortium**

**Platform:** Ovid
**Coverage:** From database inception to final date
**Limits:** English language

| **Line** | **Search terms** |
| --- | --- |
| 1 | (“mental health*” or “mental ill*” or “mental disorder*” or depress* or anxi* or suicid* or “self-harm” or “self harm” or selfharm or “self injur*”).mp. |
| 2 | (help-seeking or help seeking or helpseeking).mp. |
| 3 | ((seek* or sought or seeking) adj3 (help or support or care or treatment)).mp. |
| 4 | ((receiv* or get* or obtain*) adj3 (mental health adj3 (help or support or care or treatment))).mp. |
| 5 | (help-seeking adj3 (attitude* or intention* or behavio?r*)).mp. |
| 6 | ((“barrier*” or “facilitator*”) adj3 help-seeking).mp. |
| 7 | ((access* or engag* or contact*) adj3 (mental health adj3 (service* or care or support))).mp. |
| 8 | ((attitude* or perception*) adj5 (mental health adj5 (service* or care))).mp. |
| 9 | 2 or 3 or 4 or 5 or 6 or 7 or 8 |
| 10 | (“foster care” or “foster home care” or “foster child*” or “foster youth*”).mp. |
| 11 | (“kinship care” or “relative care” or “kinship placement*”).mp. |
| 12 | (“residential care” or “residential home*” or “children’s home*” or “group home*”).mp. |
| 13 | (“out-of-home care” or “out of home care” or “looked after” or “looked-after”).mp. |
| 14 | ((care adj2 leav*) or (care adj2 experienc*)).mp. |
| 15 | (transition* adj3 care).mp. |
| 16 | 10 or 11 or 12 or 13 or 14 or 15 |
| 17 | (“young people” or “young person*” or “young adult*” or “emerging adult*” or adolescen* or youth or youths or teen* or child*).mp. |
| 18 | 1 and 9 and 16 and 17 |

## **Social Policy & Practice**

**Coverage:** From database inception to final date

**Limits:** English language limit not available

| **Line** | **Search terms** |
| --- | --- |
| 1 | (“mental health*” or “mental ill*” or “mental disorder*” or depress* or anxi* or suicid* or “self-harm” or “self harm” or selfharm or “self injur*”).mp. |
| 2 | (help-seeking or help seeking or helpseeking).mp. |
| 3 | ((seek* or sought or seeking) adj3 (help or support or care or treatment)).mp. |
| 4 | ((receiv* or get* or obtain*) adj3 (mental health adj3 (help or support or care or treatment))).mp. |
| 5 | (help-seeking adj3 (attitude* or intention* or behavio?r*)).mp. |
| 6 | ((“barrier*” or “facilitator*”) adj3 help-seeking).mp. |
| 7 | ((access* or engag* or contact*) adj3 (mental health adj3 (service* or care or support))).mp. |
| 8 | ((attitude* or perception*) adj5 (mental health adj5 (service* or care))).mp. |
| 9 | 2 or 3 or 4 or 5 or 6 or 7 or 8 |
| 10 | (“foster care” or “foster home care” or “foster child*” or “foster youth*”).mp. |
| 11 | (“kinship care” or “relative care” or “kinship placement*”).mp. |
| 12 | (“residential care” or “residential home*” or “children’s home*” or “group home*”).mp. |
| 13 | (“out-of-home care” or “out of home care” or “looked after” or “looked-after”).mp. |
| 14 | ((care adj2 leav*) or (care adj2 experienc*)).mp. |
| 15 | (transition* adj3 care).mp. |
| 16 | 10 or 11 or 12 or 13 or 14 or 15 |
| 17 | (“young people” or “young person*” or “young adult*” or “emerging adult*” or adolescen* or youth or youths or teen* or child*).mp. |
| 18 | 1 and 9 and 16 and 17 |

## **ASSIA: Applied Social Sciences Index & Abstracts**

**Coverage:** From database inception to final date
**Limits:** English language

**S1 Mental Health**

("mental health*" OR "mental ill*" OR "mental disorder*" OR depress* OR anxi* OR suicid* OR "self-harm" OR "self harm" OR selfharm OR "self injur*") OR MAINSUBJECT.EXACT("Mental health")

**S2: Help-seeking**

(help-seeking OR "help seeking" OR helpseeking) OR (seek* NEAR/3 help) OR (access* NEAR/3 "mental health") OR (engag* NEAR/3 "mental health") OR (contact* NEAR/3 "mental health") OR (attitude* NEAR/5 "mental health service*") OR (perception* NEAR/5 "mental health service*")

**S3 Out-of-home care Context**

("foster care" OR "foster home care" OR "foster child*" OR "foster youth*") OR ("kinship care" OR "relative care" OR "kinship placement*") OR ("residential care" OR "residential home*" OR "children's home*" OR "group home*") OR ("out-of-home care" OR "out of home care" OR "looked after" OR "looked-after") OR (care NEAR/2 leav*) OR (care NEAR/2 experienc*) OR MAINSUBJECT.EXACT("Foster care") OR MAINSUBJECT.EXACT("Kinship care") OR MAINSUBJECT.EXACT("Residential care")

**S4 Young People**

("young people" OR "young person*" OR "young adult*" OR "emerging adult*" OR adolescen* OR youth OR youths OR teen* OR child*)

**Final combination:** S1 AND S2 AND S3 AND S4

## **SSCI: Social Sciences Citation Index**

**Platform:** Web of Science
**Coverage:** From database inception to final date

**Limits:** English language

**S1 Mental health**
“mental health*” OR “mental ill*” OR “mental disorder*” OR “psychological health*” OR “psychological ill*” OR “psychological disorder*” OR depress* OR anxi* OR suicid* OR “self-harm” OR “self harm” OR selfharm OR “self injur*”

**S2 Help-seeking**
help-seeking OR “help seeking” OR helpseeking OR seek* NEAR/3 help OR access* NEAR/3 “mental health” OR engag* NEAR/3 “mental health” OR contact* NEAR/3 “mental health” OR attitude* NEAR/5 “mental health service*” OR perception* NEAR/5 “mental health service*”

**S3 Out-of-home care context**
“foster care” OR “foster home care” OR “foster child*” OR “foster youth*” OR “kinship care” OR “relative care” OR “kinship placement*” OR “residential care” OR “residential home*” OR “children’s home*” OR “group home*” OR “out-of-home care” OR “out of home care” OR “looked after” OR “looked-after” OR care NEAR/2 leav* OR care NEAR/2 experienc* OR transition* NEAR/3 care

**S4 Young people**
“young people” OR “young person*” OR “young adult*” OR “emerging adult*” OR adolescen* OR youth OR youths OR teen* OR child*

**Final combination:** S1 AND S2 AND S3 AND S4

## **ProQuest Dissertations & Theses Global**

**Platform:** ProQuest
**Coverage:** From database inception to final date

**Limits:** English language

**S1 Mental health**
noft(“mental health*” OR “mental ill*” OR “mental disorder*” OR depress* OR anxi* OR suicid* OR “self-harm” OR “self harm” OR selfharm OR “self injur*”)

**S2 Help-seeking**

noft(help-seeking OR “help seeking” OR helpseeking OR seek* NEAR/3 help OR access* NEAR/3 “mental health” OR engag* NEAR/3 “mental health” OR contact* NEAR/3 “mental health” OR attitude* NEAR/5 “mental health service*” OR perception* NEAR/5 “mental health service*”)

**S3 Out-of-home care**

noft(“foster care” OR “foster home care” OR “foster child*” OR “foster youth*” OR “kinship care” OR “relative care” OR “kinship placement*” OR “residential care” OR “residential home*” OR “children’s home*” OR “group home*” OR “out-of-home care” OR “out of home care” OR “looked after” OR “looked-after” OR care NEAR/2 leav* OR care NEAR/2 experienc* OR transition* NEAR/3 care)

**S4 Young people**

noft(“young people” OR “young person*” OR “young adult*” OR “emerging adult*” OR adolescen* OR youth OR youths OR teen* OR child*)

**Final combination:** S1 AND S2 AND S3 AND S4

## **Child Development & Adolescent Studies**

**Platform:** EBSCOhost
**Coverage:** From database inception to final date

**Limits:** English-language limit not available

**S1 Mental health**
TI (“mental health*” OR “mental ill*” OR “mental disorder*” OR “psychological health*” OR “psychological ill*” OR “psychological disorder*” OR depress* OR anxi* OR suicid* OR “self-harm” OR “self harm” OR selfharm OR “self injur*” OR self-injur*) OR AB (“mental health*” OR “mental ill*” OR “mental disorder*” OR “psychological health*” OR “psychological ill*” OR “psychological disorder*” OR depress* OR anxi* OR suicid* OR “self-harm” OR “self harm” OR selfharm OR “self injur*” OR self-injur*)

**S2 Help-seeking**
TI (help-seeking OR “help seeking” OR helpseeking OR ((seek* OR sought OR seeking) N3 (help OR support OR care OR treatment)) OR ((receiv* OR get* OR obtain*) N3 (“mental health” N3 (help OR support OR care OR treatment))) OR ((access* OR engag* OR contact*) N3 (“mental health” N3 (service* OR care OR support))) OR ((attitude* OR perception*) N5 (“mental health” N5 (service* OR care))) OR ((“barrier*” OR “facilitator*”) N3 (help-seeking OR “help seeking”))) OR AB (help-seeking OR “help seeking” OR helpseeking OR ((seek* OR sought OR seeking) N3 (help OR support OR care OR treatment)) OR ((receiv* OR get* OR obtain*) N3 (“mental health” N3 (help OR support OR care OR treatment))) OR ((access* OR engag* OR contact*) N3 (“mental health” N3 (service* OR care OR support))) OR ((attitude* OR perception*) N5 (“mental health” N5 (service* OR care))) OR ((“barrier*” OR “facilitator*”) N3 (help-seeking OR “help seeking”)))

**S3 Out-of-home care**
TI (“foster care” OR “foster home care” OR “foster child*” OR “foster youth*” OR “kinship care” OR “relative care” OR “kinship placement*” OR “residential care” OR “residential home*” OR “children’s home*” OR “group home*” OR “out-of-home care” OR “out of home care” OR “looked after” OR looked-after OR ((care N2 leav*) OR (care N2 experienc*) OR (transition* N3 care))) OR AB (“foster care” OR “foster home care” OR “foster child*” OR “foster youth*” OR “kinship care” OR “relative care” OR “kinship placement*” OR “residential care” OR “residential home*” OR “children’s home*” OR “group home*” OR “out-of-home care” OR “out of home care” OR “looked after” OR looked-after OR ((care N2 leav*) OR (care N2 experienc*) OR (transition* N3 care)))

**S4 Young people**
TI (“young people” OR “young person*” OR “young adult*” OR “emerging adult*” OR adolescen* OR youth OR youths OR teen* OR child*) OR AB (“young people” OR “young person*” OR “young adult*” OR “emerging adult*” OR adolescen* OR youth OR youths OR teen* OR child*)

**Final combination:** S1 AND S2 AND S3 AND S4

## **CINAHL Plus**

**Platform:** EBSCOhost
**Coverage:** From database inception to final date
**Limits:** English language

**S1 Mental health**
(MH “Mental Health” OR MH “Depression” OR MH “Anxiety” OR MH “Self-Injurious Behavior” OR TI (“mental health*” OR “mental ill*” OR “mental disorder*” OR “psychological health*” OR “psychological ill*” OR “psychological disorder*” OR depress* OR anxi* OR suicid* OR “self-harm” OR “self harm” OR selfharm OR “self injur*” OR self-injur*) OR AB (“mental health*” OR “mental ill*” OR “mental disorder*” OR “psychological health*” OR “psychological ill*” OR “psychological disorder*” OR depress* OR anxi* OR suicid* OR “self-harm” OR “self harm” OR selfharm OR “self injur*” OR self-injur*))

**S2 Help-seeking**
(MH “Help Seeking Behavior” OR TI (help-seeking OR “help seeking” OR helpseeking) OR AB (help-seeking OR “help seeking” OR helpseeking) OR TI ((seek* OR sought OR seeking) N3 (help OR support OR care OR treatment)) OR AB ((seek* OR sought OR seeking) N3 (help OR support OR care OR treatment)) OR TI ((receiv* OR get* OR obtain*) N3 (“mental health” N3 (help OR support OR care OR treatment))) OR AB ((receiv* OR get* OR obtain*) N3 (“mental health” N3 (help OR support OR care OR treatment))) OR TI ((access* OR engag* OR contact*) N3 (“mental health” N3 (service* OR care OR support))) OR AB ((access* OR engag* OR contact*) N3 (“mental health” N3 (service* OR care OR support))) OR TI ((attitude* OR perception*) N5 (“mental health” N5 (service* OR care))) OR AB ((attitude* OR perception*) N5 (“mental health” N5 (service* OR care))))

**S3 Out-of-home care**
(MH “Child Welfare” OR MH “Foster Home Care” OR MH “Child, Foster” OR TI (“foster care” OR “foster home care” OR “foster child*” OR “foster youth*” OR “kinship care” OR “relative care” OR “kinship placement*” OR “residential care” OR “residential home*” OR “children’s home*” OR “group home*” OR “out-of-home care” OR “out of home care” OR “looked after” OR looked-after) OR AB (“foster care” OR “foster home care” OR “foster child*” OR “foster youth*” OR “kinship care” OR “relative care” OR “kinship placement*” OR “residential care” OR “residential home*” OR “children’s home*” OR “group home*” OR “out-of-home care” OR “out of home care” OR “looked after” OR looked-after) OR TI ((care N2 leav*) OR (care N2 experienc*) OR (transition* N3 care)) OR AB ((care N2 leav*) OR (care N2 experienc*) OR (transition* N3 care)))

**S4 Young people**
(MH “Adolescence” OR MH “Young Adult” OR MH “Child” OR TI (“young people” OR “young person*” OR “young adult*” OR “emerging adult*” OR adolescen* OR youth OR youths OR teen* OR child*) OR AB (“young people” OR “young person*” OR “young adult*” OR “emerging adult*” OR adolescen* OR youth OR youths OR teen* OR child*))

**Final combination:** S1 AND S2 AND S3 AND S4
