# Supplementary material for: Mental health help-seeking among young people with out-of-home care experience: a mixed-methods systematic review and meta-analysis protocol
Source: BMJ Open. 2026 Jul 20;16(7):e118618. doi: 10.1136/bmjopen-2026-118618 (PMC13386074; doi:10.1136/bmjopen-2026-118618)
Supplement: Supplementary file 3 [file bmjopen-2026-118618.reviewer_comments.pdf]

PEER REVIEW HISTORY

BMJ Open publishes all reviews undertaken for accepted manuscripts. Reviewers are asked to complete a checklist review form and are provided with free text boxes to elaborate on their assessment. These free text comments are reproduced below.

ARTICLE DETAILS

Title (Provisional)

Mental Health Help-Seeking Among Young People with Out-of-Home Care Experience: A Mixed-Methods Systematic Review and Meta-Analysis Protocol

Authors

Stander, Willem J.; Wallace, Emma Rebecca; Keemink, Jolie R.; Mohamed, Omar; Schaub, Jason

---

VERSION 1 - REVIEW

Reviewer 1

Name Connell, Catriona

Affiliation University of Stirling

Date 17-May-2026

COI I am employed at the University of Stirling within the Salvation Army Centre for Addiction Services and Research which receives part funding from The Salvation Army.

Thank you for the opportunity to review this protocol for ambitious mixed-methods systematic review on understanding mental health help-seeking among care experienced young people.

For transparency, our team recently had a review published on a similar topic in this journal, which may be helpful for you / be something you wish to critique https://bmjopen.bmj.com/content/15/6/e090349

Abstract:

Introduction could clarify why it is important that help-seeking is understood above other potentially important aspects of mental health.

Introduction:

Very well written and clear section. It would be helpful to include a clear indication of whether you consider substance use and substance use disorders as within the confines of 'mental health' or as something distinct that you are not considering. Co-morbid mental health and

substance use problems are more prevalent in this population, and adds additional complexity to help-seeking dynamics.

Methods:

Inclusion criteria overall very clear – in population you say you will include papers where 'most' participants are within the specified age range. Can you clarify what proportion you are consnidering most? 51%?

In phenomena of interest – as noted above – it would be useful to clarify whether substance use, neurodiversity, personality disturbances, and learning disability are in or out. Within help-seeking, you are including a range of elements (attitudes, decision-making, intentions and behaviours) which is not clearly followed through in the outcomes. This is a valuable distinction to make in your results.

Information sources look comprehensive, there are sizeable overlaps between EMBASE and MEDLINE, so may be worth considering if you need both, and reflecting on whether an education or nursing database may be useful? Your medline search strategy is clear, but I note it only includes depression in terms of diagnoses. You may overlook some condition specific experiences in doing so.

Extraction - a lot of information being extracted. Although it may be that your study numbers are low making this manageable. You could report the number of potentially relevant results from your pilot search to allow assessment of the feasibility of this ambitious approach

Quality appraisal – why not just use MMAT for all studies?

Synthesis – will your approach to qualitative synthesis involve coding the whole of a paper, results sections, or participant quotations?

Limitations - Add any limitations of your review methods

Dissemination – can you be more specific about of what your papers may be, if more than one.

This paper on the relationships between help-seeking and other components of healthcare access may be helpful to you https://link.springer.com/article/10.1186/1475-9276-12-18

VERSION 1 - AUTHOR RESPONSE

REVIEWER COMMENTS:

Reviewer Comments	Amendments
1. Abstract: clarify why it is important that help-seeking is understood above other potentially important aspects of mental health	We agree that the abstract should more clearly justify the focus on help-seeking. We have revised the abstract to clarify that understanding help-seeking is important because it identifies barriers and facilitators that shape whether, how and from whom care-experienced young people seek and access mental health support (see page 2, line 30-32). This focus is central to the review because elevated mental health need does not necessarily translate into timely, acceptable or accessible support, particularly where

	support pathways are mediated by carers, professionals and statutory systems.
2. Introduction: It would be helpful to include a clear indication of whether you consider substance use and substance use disorders as within the confines of 'mental health' or as something distinct that you are not considering. Co-morbid mental health and substance use problems are more prevalent in this population, and adds additional complexity to help-seeking dynamics.	We thank the reviewer for this helpful observation. We agree that substance use and substance use disorders are relevant to the mental health of care-experienced young people and may add complexity to help-seeking pathways. To clarify the scope of the review, we have revised the eligibility criteria in the Methods section. The review focuses on mental health help-seeking, in line with the funded project scope and PROSPERO registration. Studies focused solely on help-seeking for substance use, substance use disorders, or substance use treatment will therefore be excluded unless substance use is explicitly examined in relation to mental health help-seeking. Where substance use is reported as a co-occurring issue, barrier, facilitator, or contextual influence shaping mental health help-seeking, it will be extracted and considered in the synthesis (see page 9, line 255-261).
3. Methods: <u>In population</u> you say you will include papers where 'most' participants are within the specified age range. Can you clarify what proportion you are considering most? 51%?	We thank the reviewer for this helpful comment. We have revised the Population section to clarify that "most" refers to a majority of participants, defined as >50% where this can be determined. In line with Cochrane guidance, we have also specified how studies with only a subset of eligible participants, or incompletely reported age data, will be handled. Studies including wider age ranges will be eligible where data for participants aged 13–25 years can be disaggregated, or where most participants are within this age range. Where age data are incomplete, studies may still be included if they are clearly adolescent- or young-person-focused, for example through the sample description, recruitment setting, eligibility criteria, or reported median/mean age, and findings are directly relevant to mental health help-seeking (see page 8, line 237-246).<br><br>This pragmatic approach reflects known age-reporting limitations in the evidence base, including Powell et al.'s (2021) systematic review, which included adolescent-focused studies while documenting incomplete age reporting in participant characteristics. To maintain transparency, such studies will be flagged during extraction and excluded from age-specific quantitative synthesis. Where age relevance remains unclear, authors will be contacted where feasible. We

	have also clarified that studies involving carers, professionals or other adults will be eligible only where findings explicitly relate to the mental health help-seeking processes of care-experienced young people who meet, or can be reasonably assessed against, these age criteria.
4. In phenomena of interest – as noted above – it would be useful to clarify whether substance use, neurodiversity, personality disturbances, and learning disability are in or out. Within help-seeking, you are including a range of elements (attitudes, decision-making, intentions and behaviours) which is not clearly followed through in the outcomes. This is a valuable distinction to make in your results.	We thank the reviewer for this helpful request for clarification. We have revised the Exposure/phenomenon of interest section to clarify that studies focused solely on substance use, substance use disorders, substance use treatment, neurodiversity, learning disability, personality-related difficulties, educational support, disability services or behavioural management will be excluded unless mental health help-seeking is explicitly examined (see page 9, line 256-261). Where these issues are reported as co-occurring issues, barriers, facilitators or contextual influences shaping mental health help-seeking, they will be extracted and considered in the synthesis.<br><br>We have also revised the Outcomes and Data extraction sections to distinguish more clearly between help-seeking attitudes, intentions and behaviours, consistent with Rickwood and Thomas' (2012) help-seeking measurement framework and previous youth mental health help-seeking reviews (Aguirre Velasco et al., 2020; Radez et al., 2021). Recognition and appraisal of need, and decisions about whether and where to seek help, will be extracted as components of the help-seeking process rather than treated as separate outcome categories. Where possible, attitudes, intentions and behaviours will be reported separately, with appraisal, decision-making, sources of support, barriers, facilitators and access experiences synthesised alongside these domains.
5. Search Strategy - Information sources look comprehensive, there are sizeable overlaps between EMBASE and MEDLINE, so may be worth considering if you need both, and reflecting on whether an education or nursing database may be useful? Your Medline search strategy is clear, but	We thank the reviewer for these helpful comments. We have addressed each point in turn.<br><br>First, we agree that MEDLINE and EMBASE have some overlap, but have retained both because they are complementary rather than interchangeable. MEDLINE provides core biomedical, psychiatric and health-services coverage through MeSH indexing, while EMBASE provides additional biomedical, psychiatric, public health and European/international journal coverage through Emtree indexing. Given the interdisciplinary focus of this review, spanning mental

	I note it only includes depression in terms of diagnoses. You may overlook some condition specific experiences in doing so.	health, health services, child welfare and social care, searching both databases provides broader and more sensitive coverage than either database alone. Empirical studies of systematic review searching also indicate that MEDLINE alone may miss relevant studies and that combining MEDLINE and EMBASE can improve recall (Bramer et al., 2016, 2017, 2018).<br><br>Second, we agree that nursing and education/development databases may capture relevant literature not identified through the original database set. We have therefore added *CINAHL Plus* and *Child Development & Adolescent Studies* to the revised search strategy; these additional searches identified one eligible study not retrieved through the original database set (Unrau & Grinnell, 2005), which was retrieved in both databases. CINAHL Plus strengthens coverage of nursing, allied health and health-service literature, while Child Development & Adolescent Studies strengthens coverage of child development, adolescence and education-adjacent literature relevant to young people's mental health help-seeking.<br><br>We selected Child Development & Adolescent Studies as the most appropriate education/development database because the review focuses on young people's mental health help-seeking in the context of care, development, adolescence and service access, rather than education settings or educational outcomes specifically. Additional education databases available through EBSCOhost, including ERIC, British Education Index, Education Abstracts and Educational Administration Abstracts, were piloted but did not identify substantively relevant records beyond those retrieved through Child Development & Adolescent Studies and the existing interdisciplinary database set.<br><br>Third, in relation to condition-specific terms, the search strategy was designed to identify studies of mental health help-seeking rather than diagnosis-specific service use for each individual mental health condition. The strategy includes broad mental health terms, including "mental health*", "mental ill*" and "mental disorder*", alongside controlled vocabulary and free-text terms for common youth mental health presentations, including depression, anxiety, self-harm/self-injury and suicidality. The search strategy was iteratively piloted and refined in consultation with

	an information specialist to balance sensitivity and specificity, as earlier searches retrieved a high volume of low-specificity records relative to the focused review questions.<br><br>During piloting, we checked the revised strategy against Powell et al.'s (2021)previous systematic review, which represents the closest prior evidence synthesis in this topic area and focused primarily on qualitative and mixed-methods evidence relating to mental health help-seeking among young people and carers in foster and kinship care. The revised search recovered all studies included in Powell et al.'s review, providing reassurance that it retained sensitivity to the most closely aligned prior evidence base while extending coverage to quantitative evidence, residential care, care leavers and additional interdisciplinary databases.<br><br>The manuscript and supplementary search strategy file have been revised accordingly (see Supplementary Appendix 2).
6. <u>Extraction -</u> a lot of information being extracted. Although it may be that your study numbers are low making this manageable. You could report the number of potentially relevant results from your pilot search to allow assessment of the feasibility of this ambitious approach	We agree that the extraction framework is detailed; however, this is a relatively small and focused evidence base. This is supported by screening completed to date and by Powell et al.'s (2021) previous review (N = 5 included studies), which is the closest prior review in this area and included only five studies focused on mental health help-seeking among young people in foster and kinship care. Based on current screening progress, we anticipate that the final number of included studies will fall within a manageable range, approximately 15–30 studies.<br><br>The level of extraction detail is therefore considered feasible, particularly as many out-of-home care studies report limited data across some domains, including equity-relevant characteristics, as noted in the rationale for the review.<br><br>We have revised the manuscript to clarify that the extraction form will be piloted independently by two reviewers on 10 included studies and refined before full data extraction. Following piloting, data from all included studies will be extracted independently by two reviewers, with discrepancies resolved through discussion and, where necessary, consultation with a third member of the review team (see page 12, line 348-350).

7. <u>Quality appraisal –</u> why not just use MMAT for all studies?	We considered using MMAT across all included study designs. However, we have retained design-specific appraisal tools because the review uses a segregated synthesis, with qualitative and quantitative evidence synthesised separately before narrative integration. We therefore considered it more appropriate to use CASP for qualitative studies, an adapted NOS for observational quantitative studies, and MMAT for mixed-methods studies. This approach is consistent with mixed-methods review guidance (Hong & Pluye, 2019; Lizarondo et al., 2020) and published systematic reviews that have used design-specific tools across heterogenous evidence bases (e.g., De Sutter et al., 2020; Grossi et al., 2021).<br><br>We have revised the manuscript to make this rationale clearer. (page 12, line 359-361).
8. <u>Synthesis –</u> will your approach to qualitative synthesis involve coding the whole of a paper, results sections, or participant quotations?	We have revised the synthesis section to specify that qualitative synthesis will focus on the findings/results sections of included qualitative and mixed-methods studies. This will include participant quotations, author-reported themes and subthemes, interpretive commentary, and any tables or conceptual models reporting qualitative findings relevant to mental health help-seeking. We will not code the whole paper as primary synthesis data, although background, methods and discussion sections may inform contextual interpretation and quality appraisal (page 13, line 383-389).
9. <u>Limitations -</u> Add any limitations of your review methods	We thank the reviewer for this helpful comment. We have added a limitations paragraph to the manuscript. This now notes that the English-language restriction may limit relevance to non-English-speaking regions and under-represent evidence from non-Western contexts. We also note that variation in OOHC, care-leaving entitlements, mental health services and help-seeking definitions across countries may limit comparability and transferability. Finally, we clarify that anticipated heterogeneity may limit the feasibility of meta-analysis, and that equity-focused synthesis using PRISMA-Equity and PROGRESS-Plus will depend on the extent and quality of reporting in included studies (page 15, line 435-444).

10. Dissemination: Can you be more specific about of what your papers may be, if more than one.	We have revised the dissemination section to clarify that we plan to publish one peer-reviewed systematic review article. We have also clarified the planned wider dissemination outputs, including conference and webinar presentations and free, accessible digital outputs co-developed with care-experienced young people (page 15, line 446-454).

References

Aguirre Velasco, A., Cruz, I. S. S., Billings, J., Jimenez, M., & Rowe, S. (2020). What are the barriers, facilitators and interventions targeting help-seeking behaviours for common mental health problems in adolescents? A systematic review. *BMC Psychiatry*, *20*(1), 293. https://doi.org/10.1186/s12888-020-02659-0

Bramer, W. M., Giustini, D., Kleijnen, J., & Franco, O. H. (2018). Searching Embase and MEDLINE by using only major descriptors or title and abstract fields: A prospective exploratory study. *Systematic Reviews*, *7*(1), 200. https://doi.org/10.1186/s13643-018-0864-9

Bramer, W. M., Giustini, D., & Kramer, B. M. R. (2016). Comparing the coverage, recall, and precision of searches for 120 systematic reviews in Embase, MEDLINE, and Google Scholar: A prospective study. *Systematic Reviews*, *5*(1), 39. https://doi.org/10.1186/s13643-016-0215-7

Bramer, W. M., Rethlefsen, M. L., Kleijnen, J., & Franco, O. H. (2017). Optimal database combinations for literature searches in systematic reviews: A prospective exploratory study. *Systematic Reviews*, *6*(1), 245. https://doi.org/10.1186/s13643-017-0644-y

Campbell, M., McKenzie, J. E., Sowden, A., Katikireddi, S. V., Brennan, S. E., Ellis, S., Hartmann-Boyce, J., Ryan, R., Shepperd, S., Thomas, J., Welch, V., & Thomson, H. (2020). Synthesis without meta-analysis (SWiM) in systematic reviews: Reporting guideline. *BMJ*, *368*, l6890. https://doi.org/10.1136/bmj.l6890

Cosh, S. M., McNeil, D. G., Jeffreys, A., Clark, L., & Tully, P. J. (2024). Athlete mental health help-seeking: A systematic review and meta-analysis of rates, barriers and facilitators. *Psychology of Sport and Exercise*, *71*, 102586. https://doi.org/10.1016/j.psychsport.2023.102586

De Sutter, E., Zaçe, D., Boccia, S., Di Pietro, M. L., Geerts, D., Borry, P., & Huys, I. (2020). Implementation of Electronic Informed Consent in Biomedical Research and Stakeholders' Perspectives: Systematic Review. *Journal of Medical Internet Research*, *22*(10), e19129. https://doi.org/10.2196/19129

Grossi, A., Hoxhaj, I., Gabutti, I., Specchia, M. L., Cicchetti, A., Boccia, S., & de Waure, C. (2021). Hospital contextual factors affecting the implementation of health technologies: A systematic review. *BMC Health Services Research*, *21*(1), 407. https://doi.org/10.1186/s12913-021-06423-2

Gulliver, A., Griffiths, K. M., & Christensen, H. (2010). Perceived barriers and facilitators to mental health help-seeking in young people: A systematic review. *BMC Psychiatry*, *10*(1), 113. https://doi.org/10.1186/1471-244X-10-113

Hong, Q. N., & Pluye, P. (2019). A Conceptual Framework for Critical Appraisal in Systematic Mixed Studies Reviews. *Journal of Mixed Methods Research*, *13*(4), 446–460. https://doi.org/10.1177/1558689818770058

Lizarondo, L., Stern, C., Carrier, J., Godfrey, C., Rieger, K., Salmond, S., Apostolo, J., Kirkpatrick, P., & Loveday, H. (2020). Chapter 8: Mixed methods systematic reviews. In Aromataris, E., Lockwood, C., Porritt, K., Pilla, B., & Jordan, Z. (Eds), *JBI Manual for Evidence Synthesis*. JBI. https://doi.org/https://doi.org/10.46658/JBIMES-24-07

McPhail, L., Thornicroft, G., & Gronholm, P. C. (2024). Help-seeking processes related to targeted school-based mental health services: Systematic review. *BMC Public Health*, *24*(1), 1217. https://doi.org/10.1186/s12889-024-18714-4

Powell, K., Huxley, E., & Townsend, M. L. (2021). Mental health help seeking in young people and carers in out of home care: A systematic review. *Children and Youth Services Review*, *127*. Scopus. https://doi.org/10.1016/j.childyouth.2021.106088

Radez, J., Reardon, T., Creswell, C., Lawrence, P. J., Evdoka-Burton, G., & Waite, P. (2021). Why do children and adolescents (not) seek and access professional help for their mental health problems? A systematic review of quantitative and qualitative studies. *European Child & Adolescent Psychiatry*, *30*(2), 183–211. https://doi.org/10.1007/s00787-019-01469-4

Rickwood, D., & Thomas, K. (2012). Conceptual measurement framework for help-seeking for mental health problems. *Psychology Research and Behavior Management*, *5*, 173–183. https://doi.org/10.2147/PRBM.S38707

Unrau, Y. A., & Grinnell, R. M. (2005). Exploring Out-of-Home Placement as a Moderator of Help-Seeking Behavior Among Adolescents Who Are High Risk. *Research on Social Work Practice*, *15*(6), 516–530. https://doi.org/10.1177/1049731505276302

Zhao, R., Amanvermez, Y., Pei, J., Castro-Ramirez, F., Rapsey, C., Garcia, C., Ebert, D. D., Haro, J. M., Fodor, L. A., David, O. A., Rankin, O., Chua, S. N., Martínez, V., Bruffaerts, R., Kessler, R. C., & Cuijpers, P. (2025). Research Review: Help-seeking intentions, behaviors, and barriers in college students – a systematic review and meta-analysis. *Journal of Child Psychology and Psychiatry*, *66*(10), 1593–1605. https://doi.org/10.1111/jcpp.14145

VERSION 2 - REVIEW

Reviewer **1**

Name **Connell, Catriona**

Affiliation **University of Stirling**

Date **26-Jun-2026**

COI

Thank you for comprehensively addressing the clarifications from peer review. Good luck with the work.
